# A Dynamic Network Approach to the Study of Syntax

**DOI:** 10.3389/fpsyg.2020.604853

**Published:** 2020-11-23

**Authors:** Holger Diessel

**Affiliations:** Department of English, Friedrich-Schiller-Universität Jena, Jena, Germany

**Keywords:** usage-based linguistics, emergent grammar, construction grammar, network theory, syntax, domain-general processes

## Abstract

Usage-based linguists and psychologists have produced a large body of empirical results suggesting that linguistic structure is derived from language use. However, while researchers agree that these results characterize grammar as an emergent phenomenon, there is no consensus among usage-based scholars as to how the various results can be explained and integrated into an explicit theory or model. Building on network theory, the current paper outlines a structured network approach to the study of grammar in which the core concepts of syntax are analyzed by a set of relations that specify associations between different aspects of a speaker’s linguistic knowledge. These associations are shaped by domain-general processes that can give rise to new structures and meanings in language acquisition and language change. Combining research from linguistics and psychology, the paper proposes specific network analyses for the following phenomena: argument structure, word classes, constituent structure, constructions and construction families, and grammatical categories such as voice, case and number. The article builds on data and analyses presented in Diessel (2019; *The Grammar Network. How Linguistic Structure is Shaped by Language Use*) but approaches the topic from a different perspective.

## Introduction

In the usage-based approach, language is seen as a dynamic system that is shaped by domain-general processes, such as conceptualization, analogy and (joint) attention, which are not specific to language but also used in other cognitive domains, e.g., in visual perception or (non-linguistic) memory (Bates and MacWhinney, 1989; Bybee, 2010; Ibbotson, 2020; see also Diessel, 2017). Given a particular communicative intention, speakers have to make a range of linguistic decisions in order to express the intended meaning in an utterance (Levelt, 1989), and listeners have to make similar decisions in order to interpret the elements they encounter in a sentence or phrase (MacDonald et al., 1994). Domain-general processes influence both speaking and listening, which may have long-term effects on the development of linguistic structure if speakers’ and listeners’ linguistic decisions become routinized through frequency or repetition (Diessel, 2019, p. 23–39).

Frequency of language use plays a crucial role in the emergentist and usage-based study of language (see Diessel, 2007; Diessel and Hilpert, 2016 for reviews). Linguistic elements that are frequently used to express a particular communicative intention become entrenched in memory, which does not only make these elements more easily accessible in future language use but may also alter their structure and meaning: Frequent expressions are prone to undergo phonetic reduction, semantic bleaching and chunking and may develop into lexical prefabs, grammatical markers or bound morphemes (Bybee and Hopper, 2001; Bybee, 2010).

The dynamic view of linguistic structure poses new challenges to linguistic theory. In particular, it makes it necessary to reconsider the format of linguistic representations. Traditionally, linguistic representations are derived from a small set of primitive categories and rules, or constraints, that are defined prior to the analysis of any particular structure. In this approach, grammatical categories, such as noun, case and phrase, are used as “tools” for analyzing stable and discrete representations of linguistic structure (Jackendoff, 2002, p. 75). However, if we think of language as a dynamic system, there are no primitive concepts of grammatical analysis and linguistic representations are emergent and transient. I use the term “emergent” in the sense of systems theory (Thelen and Smith, 1995) for a particular type of development whereby a complex phenomenon evolves from the interaction of many parts whose accumulated properties are not sufficient to explain the holistic properties of the phenomenon they created; and I use the term “transient” for phenomena that are in principle always changing—that never really reach a fixed state.

Over the past 25 years, linguists and psychologists have produced a large body of empirical results supporting the emergentist view of linguistic structure (e.g., MacWhinney, 1999; Tomasello, 2003; Bybee, 2010). However, while researchers agree that linguistic structure is emergent and transient, they have not yet developed an explicit theory or model to explain the various findings and to generate specific hypotheses for future research. To be sure, there are some interesting proposals as two how frequency and experience shape linguistic structure and how emergent linguistic knowledge is represented in speakers’ minds. Yet, many of these proposals are too vague and general in order to provide a structured model of grammar.

For instance, some scholars have argued that exemplar theory provides a good framework for analyzing linguistic structure (e.g., Abbot-Smith and Tomasello, 2006; Bybee, 2006; Bod, 2009). On this view, all aspects of linguistic knowledge are represented by a cluster of similar tokens that reflect a language user’s experience with particular linguistic elements. Similar tokens overlap in memory and strengthen the activation value of linguistic representations, which in turn may influence their future use.

Exemplar theory has been quite successful in modeling the emergence of speech sound categories (Johnson, 1997; Bybee, 2001; Pierrehumbert, 2001); but when it comes to grammar, exemplar theory provides nothing but a crude approximation of the effect of frequency on a speaker’s linguistic knowledge. Of course, like all other linguistic elements, grammatical categories are reinforced in memory through repetition; but this is not sufficient to explain how grammatical structure is derived from language use (see Diessel, 2016 for discussion).

Grammar is a highly complex system that involves schematic representations and different types of categories that interact with each other in intricate ways. Both abstract schemas and interacting categories are difficult to explain in a pure exemplar model. In order to analyze the emergence and interaction of grammatical categories, one needs a different approach that takes into account the full range of domain-general processes (and not just exemplar learning) and that differentiates between different aspects of linguistic knowledge (e.g., semantic vs. syntactic knowledge, schematic vs. lexical knowledge) and different types of categories (e.g., word class categories, phrasal categories, grammatical relations).

In this paper, I argue that network theory (Baronchelli et al., 2013; Barabási, 2016) provides a useful framework for the analysis of grammar in the emergentist approach (see Bates and MacWhinney, 1989 for an early network model of grammar). Network theory is based on mathematical graph theory and has been used by researchers from various disciplines to investigate a wide range of phenomena including electric power systems, economical systems, traffic systems, social relationships, the brain and the World Wide Web (Buchanan, 2002; Sporns, 2011; Barabási, 2016). Like exemplar theory, network theory can explain emergent phenomena; but the network approach is much more powerful than the standard model of exemplar theory.

The basic structure of a network model is simple. All network models consist of two basic elements: (i) nodes, also known as vertices, and (ii) connections, also known as links, arcs or relations. However, there are many different types of network models with different architectures, different mechanisms of learning and change, and different measurements for the emergence of structure (Barabási, 2016), making network theory a very powerful instrument for analyzing complex (adaptive) systems such as a person’s linguistic knowledge.

Network models are widely used by cognitive scientists to analyze the mental lexicon (see Siew et al., 2019 for a recent review) and have also been invoked by usage-based linguists to explain certain grammatical phenomena such as morphological paradigms (Bybee, 1995; Hay and Baayen, 2005) and the taxonomic organization of constructions (Goldberg, 1995; Hilpert, 2014). However, while these accounts have shed new light on some aspects of linguistic structure, grammatical categories have hardly ever been analyzed within a network model (but see Croft, 2001). In fact, although usage-based linguists agree that grammatical categories are emergent and transient, in practice, they often use them as predefined concepts, similar to the way grammatical categories are used in the “toolkit” approach (Jackendoff, 2002, p. 75).

Challenging this practice, the current paper argues that grammatical categories, such as noun, noun phrase and case, are best analyzed in the framework of a structured network model in which all grammatical concepts are defined by particular types of links or relations that specify associations between different aspects of a speaker’s linguistic knowledge. The approach is inspired by connectionism (Elman et al., 1996; Chang et al., 2006) and draws on research in morphology (Bybee, 1995; Hay, 2003) and construction grammar (Croft, 2001; Bybee, 2010; see also Diessel, 1997, 2015). However, it differs from all previous accounts in that it proposes a specific network architecture for the analysis of particular grammatical concepts. Concentrating on some of the most basic concepts of syntax, this paper considers the following phenomena:

1. Constructions
2. Argument structure
3. Word classes
4. Constituent structure
5. Grammatical categories such as voice, case and number
6. Construction families

As we will see, all of these phenomena can be analyzed as dynamic networks shaped by domain-general processes of language use. The paper builds on ideas presented in Diessel (2019), but these ideas will be discussed from a different perspective and in light of other data. We begin with one of the most basic concepts of usage-based research on grammar, i.e., the notion of construction.

## Constructions

In accordance with many other researchers, I assume that linguistic structure consists of constructions that combine a particular form with meaning (Goldberg, 1995, p. 5). However, contrary to what is sometimes said in the literature, constructions are not primitive units, as, for instance, suggested by Croft (2001):

Constructions, not categories and relations, are the basic, primitive units of syntactic representation (Croft, 2001, p. 46).

I agree with Croft that syntactic categories (e.g., noun, verb) and grammatical relations (e.g., subject, object) are non-basic and derived; but I disagree with the claim that constructions are basic and primitive. It is not entirely clear what Croft means with this, but contrary to what the above quote suggests, I maintain that constructions are emergent and transient like all other aspects of linguistic structure. Specifically, I claim that one can think of constructions as networks that involve three different types of associative relations: (i) symbolic relations, connecting form and meaning, (ii) sequential relations, connecting linguistic elements in sequence, and (iii) taxonomic relations, connecting linguistic representations at different levels of abstraction (Diessel, 2019, p. 41–112; see Schmid, 2020 for a related proposal).

### Taxonomic Relations

Taxonomic relations have been at center stage in construction grammar since its beginning (Goldberg, 1995, p. 72–77). It is a standard assumption of usage-based construction grammar that linguistic structure is represented at different levels of schematicity that are connected by taxonomic or inheritance relations, as illustrated in example (1).(1)

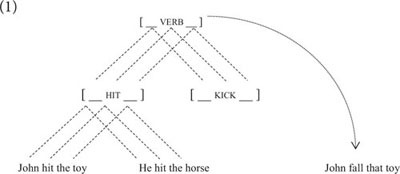

One piece of evidence for the existence of constructional schemas and constructional inheritance comes from overgeneralization errors, such as *John fall that toy*, in L1 acquisition (Bowerman, 1988). Assuming that the ambient language only includes intransitive uses of the verb *fall*, the transitive use suggests that this child must have acquired a transitive schema in order to use *fall* as a transitive verb (for a recent discussion of overextension errors of argument-structure constructions in L1 acquisition see Diessel, 2013; see also Brooks et al., 1999).

Schematic representations of linguistic structure emerge as generalizations over lexical sequences with similar forms and meanings. While this can happen at any time, the basic constructions of a language are learned during early childhood. There is a large body of research on schema extraction in infancy (e.g., Gómez and Gerken, 1999; Gómez, 2002; Gerken, 2006; see Frost et al., 2019 for a recent review) and the acquisition of argument-structure constructions during the preschool years (e.g., Tomasello and Brooks, 1998; Brooks and Tomasello, 1999; see Diessel, 2013 for a review). The emergence of constructional schemas involves a wide range of cognitive processes, but in particular, it involves categorization and analogy, which are crucially influenced by similarity and type and token frequency (Tomasello, 2003; Bybee, 2010).

### Sequential Relations

Language unfolds in time and all linguistic elements are arranged in linear or sequential order. The sequential arrangement of linguistic elements is motivated by semantic and pragmatic factors, such as the given-before-new principle (Chafe, 1994) and iconicity of sequence (Diessel, 2008). Yet, linguistic elements that are frequently used together become associated with each other, regardless of any semantic or pragmatic considerations. This is reflected in the emergence of lexical chunks, or lexical prefabs, that are bound together by sequential links or relations (Wray, 2002; Arnon and Snider, 2010; Lorenz and Tizón-Couto, 2017).

Sequential links are the result of automatization, which is a well-known process of human cognition (Logan, 1988) that does not only concern language but also non-linguistic phenomena such as counting and dancing (Ghilardi et al., 2009). Sequential links have an inherent forward orientation as evidenced by the fact that the speech participants are usually ahead of the speech stream. This has been a hotly debated topic of recent research in psycholinguistics (Altmann and Kamide, 1999; Levy, 2008; Kuperberg and Jaeger, 2016). There is plenty of evidence that speech participants “predict” upcoming elements in an unfolding sentence or discourse (Kamide et al., 2003; Fine et al., 2013).

Since automatization is driven by frequency of occurrence, sequential relations are weighted. All else being equal, the more frequently a linguistic string is processed, the stronger the sequential links between its component parts. This holds for both lexical strings and schematic processing units or constructional schemas (cf. 2). Both are organized in “chunk hierarchies” (Gobet et al., 2001) that reflect the combined effect of conceptual factors and automatization.(2)

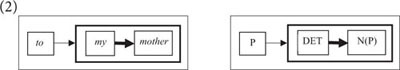

### Symbolic Relations

Finally, symbolic relations are associations between form and meaning. Following de Saussure (1916), the pairing of form and meaning, or signifier and signified, is commonly interpreted as a linguistic sign. In the literature, linguistic signs are usually characterized as stable concepts; but if we look at the development of linguistic signs in acquisition and change, we see that symbolic associations are emergent and gradient, just like all other associative connections of the language network. Specifically, I claim that symbolic relations arise from recurrent paths of semantic interpretation that become entrenched and conventionalized through repetition and social interaction (Diessel, 2019, p. 90–112).

The construction-based literature has emphasized the parallels between lexemes and constructions (Goldberg, 1995; Croft, 2001; Hilpert, 2014). Both are commonly defined as signs or symbols; but while one might think of constructions as symbolic entities, it is important to recognize that the conceptual processes involved in the semantic interpretation of constructions are distinct from those of lexemes.

In cognitive psychology, lexemes are commonly characterized as cues or stimuli that do not represent meaning but serve to evoke a particular interpretation (Barsalou, 1999; Elman, 2009). Every lexeme is interpreted against the background of an entire network of conceptual knowledge. The lexeme “sky,” for instance, designates an area above the earth that is associated with a wide variety of concepts including “sun,” “cloud,” “rain,” “bird,” “flying,” “blue,” “thunder,” and “heaven” (cf. 3). Since the concept of “earth” is entailed in the meaning of “sky,” it is generally activated as its conceptual base. Yet, the activation of all other concepts varies with the context.(3)

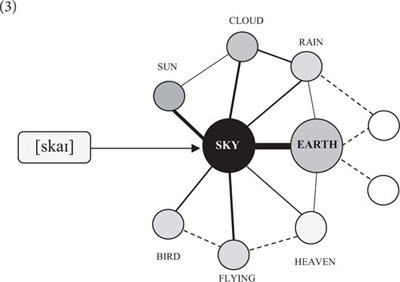

Psychologists refer to this as “spreading activation” (Collins and Loftus, 1975; Anderson, 1983; Dell, 1986). On this account, lexemes provide access to a figure node, or figure concept, of an association network from where it spreads to related background nodes or background concepts. The best piece of evidence for spreading activation comes from lexical priming (Tulving and Schacter, 1990; Hoey, 2005). When people are given a word prior to a lexical decision task, they respond faster to semantically and/or phonetically related items than to unrelated words.

Like lexemes, constructions provide cues for the creation of meaning, but the conceptual processes evoked by constructions are distinct from those of lexemes. Constructions are linear processing units that emerge as generalizations over lexical sequences with similar forms and meanings. Since (schematic) constructions abstract away from particular lexical units, they do not directly tap into world knowledge (like lexical items). Rather, constructions provide processing instructions as to how the concepts evoked by a string of lexemes are integrated into a coherent semantic interpretation. Argument structure constructions, for instance, instruct the listener to assign particular semantic roles (e.g., agent, recipient, theme) to certain lexical expressions (cf. 4). Thus, contrary to what is commonly assumed in the construction-based literature, I submit that, while constructions are meaningful, the semantic processes evoked by constructions are crucially distinct from those evoked by lexemes (Diessel, 2019, p. 107–112; see also Chen, 2020 for a recent network-based approach to the study of constructional semantics).(4)

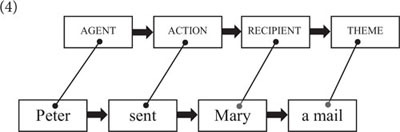

In sum, constructions are not basic or primitive units. Rather, constructions can be seen as dynamic networks that involve taxonomic, sequential and symbolic relations. Each one of these relations is shaped by an intricate interplay of several cognitive processes including conceptualization, analogy, categorization, pragmatic inference, automatization and social cognition. Together, the three relations define constructions as emergent and transient concepts. Crucially, these concepts interact in complex ways at a higher-level network where linguistic elements are organized in syntactic categories and paradigms. In order to analyze this higher-level network, I propose two further types of relations: (i) filler-slot relations, which specify associations between the slots of constructional schemas and lexical or phrasal fillers, and (ii) constructional relations, which specify associations between constructions at the same level of abstraction^1^. In what follows, I argue that these relations are crucial to the analysis of various grammatical phenomena including argument structure, word classes, phrase structure, grammatical categories such as voice, case and number, and construction families.

## Argument Structure

Traditionally, argument structure is determined by verbs (Levin and Rappaport Hovav, 2005), but in construction grammar, argument structure is not just a matter of verbs but also of constructions (Goldberg, 1995). Verbs select a set of participant roles and argument-structure constructions provide slots for certain semantic types of participants. If a verb and a construction specify the same participant roles, they are semantically compatible with each other and may fuse. This is, in a nutshell, Goldberg’s Semantic Coherence Principle (Goldberg, 1995, p. 50), which has been very influential in the constructivist approach to the analysis of argument structure. However, this principle is not without problems. As I see it, there are two general problems that can be easily resolved if we think of argument structure as a network.

The first problem is that there are many idiosyncrasies. In Goldberg’s theory, fusion is a matter of semantic compatibility, but very often fusion is not semantically motivated. Take, for instance, the double-object construction (*She gave her friend a present*), which denotes an act of transfer and typically occurs with transfer verbs, e.g., *give, send, offer, bring*. Most of these verbs also appear in the *to*-dative construction (*She gave a present to her friend*), but there are various idiosyncrasies. *Donate* and *say*, for instance, designate transfer—physical or communicative transfer—like *give* and *tell*; yet, unlike *give* and *tell*, *donate* and *say* occur only in the *to*-dative construction (*She donated some money to the Red Cross; He said no to her*) but not in the double object construction (*^∗^She donated the Red Cross some money; ^∗^He said her no*). Conversely, there are verbs such as *forgive* and *envy* that may occur in the double-object construction (*She forgave him his faults; I envy you your car*), although these verbs do not denote any obvious sense of transfer (Goldberg, 1995, p. 130).

Goldberg is aware of these idiosyncrasies and considers them “exceptions” (Goldberg, 1995, p. 129–132); but since lexical inconsistencies of this type are very common, some scholars have questioned the importance of high-level schemas for the analysis of argument structure. In particular, Boas (2003, 2008) has argued that argument-structure constructions are organized around particular verbs, or narrow verb classes, and that fully schematic constructions are only of minor importance to the analysis of argument structure (see also Faulhaber, 2011).

A related problem is that current theories of argument structure do not account for the statistical asymmetries in the distribution of individual verbs. As many corpus linguists have pointed out, verbs and constructions are skewed in their distribution. *Give*, for instance, is more frequent in the double-object construction than statistically expected and less frequent than expected in the *to*-dative construction; but for *bring* it is the other way around (Gries and Stefanowitsch, 2004).

Lexical idiosyncrasies and asymmetries have also been noted with regard to many other types of argument-structure constructions. Consider, for instance, the active-passive alternation. Most transitive verbs can appear in both active and passive voice, but in some languages, the active-passive alternation is not fully productive. German, for example, has a number of transitive verbs (i.e., verbs selecting an accusative object) that do not occur in passive voice, e.g., *kennen* “to know,” *wissen* “to know,” *besitzen* “to own,” *kosten* “to cost,” *bekommen* “to get” (Eisenberg, 2004, p. 128–130). In English, most transitive verbs can be passivized (a notable exception is the main-verb use of *have*, see below); but there are statistical biases in the distribution of individual verbs. For example, the verbs *get*, *want* and *do* occur with a higher frequency ratio of active/passive uses than one would expect if the co-occurrence of verbs and constructions was random; but for the verbs *use*, *involve* and *publish* it is the other way around: They are biased to appear in passive voice (Gries and Stefanowitsch, 2004, p. 109).

Both the item-specific constraints on the occurrence of individual verbs and the distributional asymmetries in the co-occurrence of particular verbs and argument-structure constructions are motivated by general conceptual and discourse-pragmatic factors (e.g., Pinker, 1989; Goldberg, 1995). Nevertheless, they are not strictly predictable from these factors. There are, for instance, no obvious semantic or pragmatic reasons why the main-verb use of *have*, meaning “to own” or “to possess,” cannot be passivized given that the verbs *own* and *possess* are frequently used in passive voice (e.g., *The farm was owned by a wealthy family; He was possessed by a devil*); and there is also no obvious semantic or pragmatic reason why the English verb *know* can appear in passive voice while its German counterparts *wissen* and *kennen* are banned from the passive construction.

Taken together, these findings suggest that speakers “know” how individual verbs are used across argument-structure constructions, on top of any semantic or pragmatic factors that may motivate their use in a particular construction. Considering these findings, I suggest that argument structure is best analyzed in the framework of a dynamic network model in which verbs and constructions are related by filler-slot associations that are determined by two general factors: (i) the semantic fit between lexemes and constructions (i.e., Goldberg’s Semantic Coherence Principle), and (ii) language users’ experience with particular co-occurrence patterns (cf. 5) (see Diessel, 2019, p. 121–141 for a more detailed account).(5)

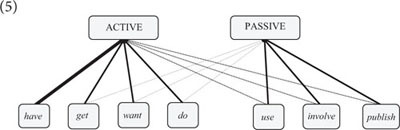

Good evidence for this hypothesis comes from psycholinguistic research on sentence processing. For instance, Trueswell (1996) showed that the processing difficulty of (reduced) passive relatives varies with the frequency with which individual verbs occur in passive voice. Since a verb such as *consider* is much more frequent in the passive than a verb such as *want* (Gries and Stefanowitsch, 2004, p. 109), passive relatives including *consider* cause significantly fewer processing problems in comprehension experiments than passive relatives including *want* (cf. 6a–b).

(6) a. The secretary (who was) *considered* by the committee was …b. The director (who was) *wanted* by the agency was …

Similar effects have been observed in psycholinguistic research with other types of constructions and other verbs (e.g., MacDonald et al., 1994; Spivey-Knowlton and Sedivy, 1995; Garnsey et al., 1997), supporting the hypothesis that speakers’ knowledge of argument-structure constructions includes filler-slot associations between individual verbs and the verb slots of particular constructions.

## Word Classes

The same network approach can be applied to grammatical word classes and phrase structure (Diessel, 2019, p. 143–171, 191–195). Traditionally, word class categories are seen as properties of lexical items (e.g., *tree* is a “noun”), but one can also think of word classes as slots of constructional schemas. Consider, for instance, the contrast between nouns and verbs in English. There are morphological, phrasal and clausal constructions including noun slots and verb slots, to which I refer as N/V-schemas (cf. 7a–c).(7)

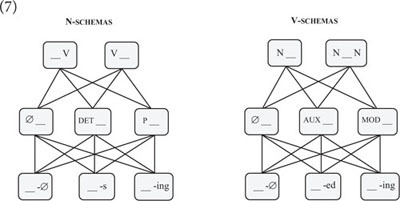

Following Croft (1991, p. 36–148) and Langacker (1991, p. 59–100), I assume that word class schemas give rise to particular conceptualizations of lexical expressions in order to use these expressions for particular speech act functions. N-schemas conceptualize the content of a lexeme as a non-relational and a-temporal entity that is used to perform an act of reference; whereas V-schemas conceptualize the content of a lexeme as a relational and temporal entity that is used to perform an act of predication. The lexeme *fax*, for instance, refers to an entity if it occurs in an N-schema (cf. 8a), and it designates a process if it occurs in a V-schema (cf. 8b).

(8) a. John sent me a *fax*.b. John *faxed* me a message.

N/V-schemas attract particular semantic types of lexical items: items that designate an entity such as the word *table* typically occur in N-schemas; whereas items that designate an action, such as the word *drink*, tend to occur in V-schemas (see Croft, 1991, p. 87–93 for quantitative corpus data from several languages supporting this analysis). However, crucially, while the co-occurrence of lexemes and word class schemas is semantically motivated, this is not just a matter of semantics but also of experience. Speakers “know,” for example, that a word such as *crime* is exclusively used in N-schemas despite the fact that *crime* designates an action, and they also “know” that *table* and *drink* appear in both N-schemas and V-schemas despite the fact that *table* (in its basic use) designates an entity and *drink* an action. In other words, speakers associate particular lexemes with specific word class schemas and the strength of these associations is again determined by two factors: the semantic fit between lexemes and schemas, and language users’ experience with particular co-occurrence patterns (cf. 9).(9)

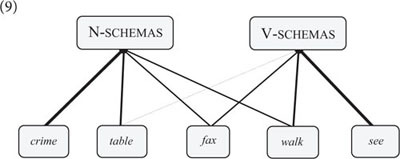

The network approach to nouns and verbs can be extended to other word classes and subclasses (Diessel, 2019, p. 157–171). Count nouns and mass nouns, for instance, are expressed by different types of N-schemas. In English, count noun schemas construe an item as a bounded entity (e.g., *That’s a cake)*, whereas mass noun schemas construe an item as an unbounded substance (e.g., *I like cake*) (Talmy, 2000, p. 50–55). Both schemas are associated with alternating and non-alternating lexemes (cf. 10). *Cake*, for instance, is an alternating lexeme, whereas *cat* is non-alternating (e.g., *That’s a cat* vs. ^∗^*I love cat*).(10)

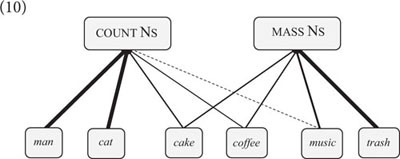

The associations are semantically motivated and entrenched by frequency of language use, but speakers can create novel connections, as in the oft-cited example *There was cat all over the driveway*, which nicely illustrates that the English mass noun schema evokes a particular conceptualization if it is applied to a new item (Langacker, 2008, p. 128–132).

Note that while word class categories are defined by semantically motivated filler-slot relations, they are also influenced by formal considerations. For example, speakers of English associate particular verb forms with particular past tense schemas based on their phonetic properties (Bybee and Slobin, 1982), which is readily explained by filler-slot relations. To illustrate, the vast majority of English verbs form the past tense by adding the *-ed* suffix (e.g., *walk → walked*). However, given a nonce verb such as *spling*, speakers may create the past tense form *splang* based on the phonetic similarity between *spling* and certain “irregular” verbs such as *sing* that form the past tense by changing the vowel [I] to [æ] (cf. 11) (Bybee and Modor, 1983).(11)

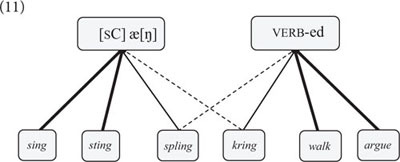

The formation of the English past tense has been a showcase for the power of the network approach in early research in connectionism (Rumelhart and McClelland, 1986). However, if we think of nouns and verbs in terms of networks (as in 11), the same approach could also be used to model the emergence of grammatical categories, if the input nodes and output nodes of a (neural) network are specified for certain conceptualizations and speech act functions.

Finally, the network approach sheds new light on cross-linguistic aspects of word classes. Most European languages have roots that are categorically linked to N-schemas or V-schemas (English is unusually flexible in this regard). However, there are other languages like Nootka (Jakobsen, 1979) and Mundari (Evans and Osada, 2005) in which lexical roots are linked to both N-schemas and V-schemas with almost no restrictions (cf. 12).(12)

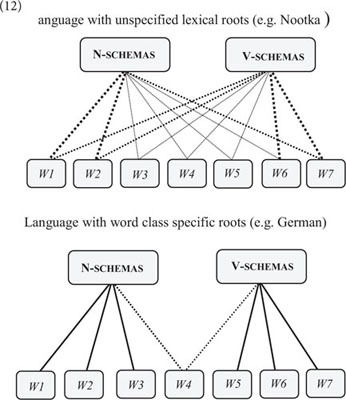

This has led some researchers to argue that languages like Nootka and Mundari do not distinguish between nouns and verbs (e.g., Jelinek, 1995), but this claim is potentially misleading as it restricts the analysis of grammatical word classes to “lexical nouns and verbs.” While lexical roots are categorically unspecified in Nootka and Mundari (with some minor restrictions; Jakobsen, 1979), there is no doubt that these languages have formally distinct N/V-schemas in which lexical roots are used as nouns and verbs for reference and prediction (Croft, 2001). Recent research in typology has questioned the existence of language universals, including the existence of universal word classes (Evans and Levinson, 2009). However, the distinction between N-schemas and V-schemas appears to be a universal trait of language that is foundational to the cognitive and linguistic organization of grammar (see Diessel, 2019, p. 152–161 for discussion).

## Constituent Structure

Like word classes, constituent structure involves filler-slot relations. The best evidence for the traditional toolkit approach comes from the analysis of syntactic constituents (Jackendoff, 2002). In generative grammar, syntactic constituents are discrete building blocks that are combined to larger structures by a set of phrase structure rules (in older versions of generative grammar) or a single syntactic operation called “merge” (in recent versions of generative grammar). The resulting structures are commonly represented in phrase structure graphs consisting of nodes and arcs that could be interpreted as some kind of network (Diessel, 2019, p. 172–173).

However, while phrase structure graphs bear some resemblance with network models, the traditional approach to the study of constituent structure is not consistent with the emergentist view of grammar in the usage-based approach. If we think of grammar as an emergent phenomenon, we need a more dynamic model of grammar that explains how constituent structure is derived from language use.

In what follows, I argue that traditional phrase structure graphs can be re-analyzed as dynamic networks of interrelated constructions. In order to understand the dynamics of these networks, one has to consider both the processes that give rise to syntactic constituents and the processes that explain how the various phrasal constituents are related.

### Phrasal Constructions

In the usage-based approach, syntactic constituents are emergent constructions that are shaped by the interaction between two cognitive processes: conceptualization and automatization. To begin with, phrasal constructions are semantically motivated by general conceptual factors. As Langacker (1997) and others have pointed out, syntactic constituents such as NP, VP, and PP are organized around relational terms that entail, or select, other types of linguistic expressions (notably pronouns and nouns). Verbs, for instance, designate actions or events that entail particular participants (see above), and adjectives designate properties that entail particular referring terms (e.g., *furry* entails an animal). Like verbs and adjectives, most grammatical function words select certain types of co-occurring expressions. Prepositions, for instance, denote semantic relations that entail nominal expressions, and auxiliaries designate temporal, aspectual or modal concepts that entail a co-occurring verb.

Since phrasal categories are organized around relational terms, they (usually) form coherent conceptual groups that may be expressed as separate intonation units (Chafe, 1994). However, while syntactic constituents are semantically motivated, they are also influenced by other factors, notably by frequency or automatization. As Bybee (2002, p. 220) notes, “the more often particular elements occur together, the tighter the constituent structure.”

In the unmarked case, conceptualization and automatization reinforce each other, but they can also be in conflict with each other. For instance, although auxiliaries are conceptually related to a co-occurring verb, the English auxiliaries *have, be* and *will* are often prosodically bound to a preceding pronoun (e.g., *I’ve, she’s, we’ll*) rather than a subsequent verb. Since the occurrence of contracted auxiliaries correlates with the joint (or transitional) probability of a pronoun and an auxiliary (Krug, 1998; Barth and Kapatsinski, 2017), it seems reasonable to assume that frequent strings such as *I’ve, she’s*, and *we’ll* are stored and processed as lexical chunks, or lexical constituents, that deviate from canonical phrase structure groups (Bybee and Scheibman, 1999).

Similar mismatches between syntactic constituents and lexical phrases have been observed with other types of expressions. Articles, for instance, are conceptually related to nominal expressions, but in German and French they are often grouped together with a preceding preposition, rather than a subsequent noun, as evidenced by the fact that these languages have developed a new set of contracted forms such as German *zum* (from *zu dem* “to the.DAT”) and French *au* (from *à le* “to the.M”).

Both conceptualization and automatization are domain-general processes (Diessel, 2019, p. 23–29). Since automatization is driven by frequency of language use, the strengthening effect of automatization varies on a scale (though this scale may not be linear). As a consequence of this (and the above described interaction between automatization and conceptualization), constituent structure is gradient and much more diverse and lexically particular than commonly assumed in traditional phrase structure analysis.

### Filler-Slot Relations

Like lexemes, phrasal constituents are associated with particular slots of constructional schemas that can be modeled by filler-slot relations. The transitive construction, for instance, includes two slots for nominal constituents functioning as subject and object or agent and theme (cf. 13).

(13) [The man]_*SUBJ*_ saw [the woman]_*OBJ*_.

In traditional phrase structure grammar, the slots of argument-structure constructions can be filled by any kind of NP, but there are well-known asymmetries between subject and object fillers. The subject slot of the transitive construction, for example, is usually filled by definite expressions, pronouns or definite NPs, that tend to be shorter and higher on the animacy scale than object NPs. Functional linguists have pointed out that the asymmetries between subject and object fillers are semantically and pragmatically motived by the meaning of the (transitive) verb and the discourse context (Chafe, 1994). However, a number of recent studies have argued that, apart from any semantic or pragmatic motivations, speakers associate certain types of phrasal fillers with certain structural positions. Good evidence for this hypothesis comes from psycholinguistic research on subject and non-subject relative clauses (cf. 14a–b).

(14) a. The student (who) *the teacher* met … NON-SUBJECT RELATIVEb. The student (who) met *the teacher* … SUBJECT RELATIVE

There is abundant evidence that non-subject relatives are more difficult to process than subject relative clauses (i.e., relatives in which the head noun functions as subject of the relative clause) (see Gordon and Lowder, 2012 for a review). Yet, the processing load of non-subject relatives varies with the type of argument fillers they include. In early psycholinguistic research on relative-clause processing, the experimental stimuli of relative clauses were usually formed with full lexical NPs (as in 14a–b), but recent research has shown that the processing load of non-subject relatives is greatly reduced if they include a pronominal subject rather than a lexical NP (cf. 15a–b) (e.g., Roland et al., 2012).

(15) a. The client (who) *the lawyer* talked to …b. The client (who) *he* talked to …

Some researchers explain the faciliatory effect of pronominal subjects on the processing of non-subject relatives by discourse factors such as topicality or givenness. According to Fox and Thompson (1990), non-subject relatives serve to “ground” the noun they modify by relating it to a “given” relative-clause subject (see also Fox and Thompson, 2007). In accordance with this hypothesis, several experimental studies have shown that pronominal subjects denoting a familiar or given referent facilitate the processing of non-subject relatives compared to relative constructions with lexical subjects denoting a new or unfamiliar subject (e.g., Mak et al., 2006; Roland et al., 2012).

However, in addition to discourse factors, such as topicality or givenness, relative-clause processing is influenced by language users’ experience with particular argument fillers (Reali and Christiansen, 2007). In corpora, non-subject relatives typically include personal pronouns as subjects, notably, first and second person pronouns are very frequent. In the Switchboard corpus, for example, *I* and *you* account for more than 80% of all subjects of non-subject relative clauses (Roland et al., 2007; see also Fox and Thompson, 2007). Building on this finding, Reali and Christiansen (2007) conducted a series of self-paced reading experiments comparing the processing of subject and non-subject relatives with certain types of subject and object fillers, as illustrated with the pronoun *you* in (16a–b).

(16) a. The consultant that *you* called emphasized the need for additional funding.b. The consultant that called *you* emphasized the need for additional funding.

In accordance with their experimental hypothesis, these researchers found that reading times correlate with the relative frequency of individual pronouns (and nouns) in subject and non-subject relatives in a very large corpus. While subject relatives are usually read faster than non-subject relatives, the relationship is reverse when argument slots are filled by pronouns that are frequent in non-subject relatives and infrequent in subject relative clauses (i.e., first and second person pronouns). Considering this finding, Reali and Christiansen argue that their “results point toward the need for a model that includes statistical information as a factor” in addition to “discourse constraints” (Reali and Christiansen, 2007, p. 18). Consistent with this view, we may propose a network model in which particular types of argument fillers are probabilistically associated with the argument slots of subject and non-subject relatives, as shown in (17).(17)

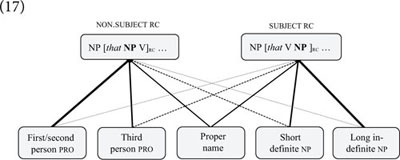

That speakers associate particular types of referring terms with particular slots of constructional schemas has also been proposed in research on the *to*-dative and double-object constructions (Bresnan et al., 2007; Bresnan and Ford, 2010) and the genitive alternation (Szmrecsanyi and Hinrichs, 2008; Wolk et al., 2013). What all of these studies have found is that the processing of syntactic structures is predictable from their relative frequency in large corpora, indicating that speakers’ syntactic knowledge of constituent structure is crucially influenced by their experience with particular constructional schemas and phrasal or lexical fillers (cf. Diessel, 2019, p. 191–195).

## Paradigmatic Alternatives: Voice, Case, Number, and Negation

In the three previous sections, we have been concerned with filler-slot relations. In the remainder of this paper, we will consider constructional relations, which specify associations between constructions at the same level of abstraction. Constructional relations have long been ignored in usage-based construction grammar, but a number of recent studies have argued that constructional relations, also known as lateral or horizontal relations, are key to understand grammatical phenomena (e.g., Diessel and Tomasello, 2005; van Trijp, 2010; Van de Velde, 2014; Norde and Morris, 2018).

Constructional relations can be divided into two basic types: (i) relations of similarity, which constitute construction families, and (ii) relations of contrast, which constitute paradigmatic alternatives of grammatical categories such as voice, case and number (Diessel, 2019, p. 199–248). We begin with the latter.

Paradigmatic alternatives are related constructions, such as active and passive sentences or singular and plural nouns, that are commonly seen as members of particular grammatical categories such as voice and number. In formal syntax, paradigmatic alternatives have been analyzed in terms of syntactic or morphological derivations. Construction grammar has abandoned the idea that linguistic structures are derived from one another or from underlying representations. Nevertheless, like any other grammatical theory, construction grammar must account for alternating categories such as active and passive voice.

If we think of grammar as a network, paradigmatic alternatives constitute pairs of horizontally related constructions. Crucially, one of the alternating categories typically serves as the default. For instance, in the case of voice, the active construction functions as the default: active sentences are more frequently used than passive sentences (Biber, 2006) and occur within a wider range of contexts (Weiner and Labov, 1983). Moreover, the linguistic encoding of active and passive sentences is asymmetrical. As it turns out, across languages, passive constructions are often marked by an extra morpheme, as illustrated by example (18b) from Sre (Mon-Khmer, Vietnam), in which the passive verb is marked by a particular passive prefix. Note, also, that in addition to the passive prefix, the agent of a passive sentence in Sre is marked by a preposition that does not occur in the corresponding active construction (cf. Engl. *This letter* ***was*** *written* ***by*** *John*).

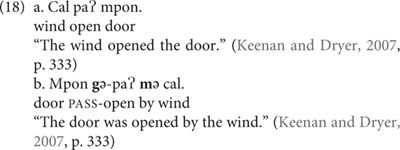

Encoding asymmetries of this type also occur with many other grammatical categories including number (*car* vs. *car-****s***), tense (*walk* vs. *walk-****ed***), aspect (*go* vs. ***is*** *go-****ing***), case (*car* vs. *car’****s***), degree (*beautiful* vs. ***more*** *beautiful*) and polarity (*He is lazy* vs. *He is* ***not*** *lazy*). Linguistic typologists refer to these asymmetries as structural markedness (Croft, 2003; see also Greenberg, 1966). Markedness is an important concept of grammar that is readily explained within a network model (Diessel, 2019, p. 223–248).

Since the occurrence of an extra morpheme correlates with frequency of language use, it has been argued that the encoding asymmetries of grammatical categories are shaped by domain-general processes of language use (Haspelmath, 2008; Haspelmath et al., 2014). Specifically, we may hypothesize that frequency of language use gives rise to particular linguistic expectations. To simplify, all else being equal, listeners expect speakers to use the more frequent member of an alternating pair of constructions. Yet, if, for whatever reason, the less frequent member is used, speakers may find it necessary to indicate their choice of construction by an extra morpheme (Kurumada and Jaeger, 2015). The best example for this is perhaps the alternation of polarity constructions. Since the majority of sentences are affirmative, negative sentences usually include a negative marker (cf. 19).(19)

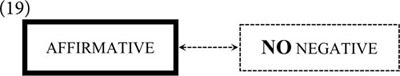

This strategy of morphological flagging is arguably the driving force behind the emergence of structural markedness (Diessel, 2019, p. 223–248). The default construction is often “zero-coded” (Haspelmath, 2006, p. 30), whereas the less frequent member takes an extra morpheme (cf. 20).(20)

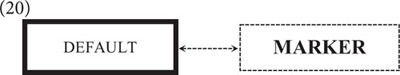

This does not only hold for syntactic constructions, such as active and passive sentences, but also for morphological constructions including inflectional categories such as number and case. Consider, for instance, the following forms of the noun *pa. t. ti* meaning “dog” in (21) from Malayalam (Dravidian, India).

(21) pa. t. ti “dog.NOM.SG”pa. t. ti-ye “dog.ACC.SG”
  pa. t. ti-ka. l “dog.NOM.PL”
  pa. t. ti-ka. l-e “dog.ACC.PL”

In Malayalam, nouns are inflected for number and case, which is usually described as a morphological paradigm consisting of a lexical root and a set of inflectional affixes. However, in construction grammar, each word form constitutes a construction in which the root is stored and processed together with a sequentially related affix (or string of affixes). The various word forms constitute a network that reflects language users’ experience with individual members of the paradigm (cf. 22).(22)

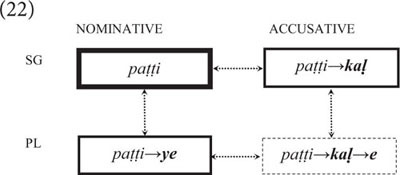

As can be seen, the various word forms differ in terms of frequency (as indicted by the strength of the boxes), which correlates with the occurrence of grammatical markers. The most frequent word form is nominative singular, which is formally unmarked, as it functions as the default. All other word forms carry at least one extra marker (for number or case), and plural nouns in accusative case take two markers (for both number and case), as they are the least frequent and least expected member of the paradigm.

What this example shows is that every construction has a particular “ecological location” in the grammar network that is defined by its relationship to other constructions in the system (Diessel, 2019, p. 223–248). This does not only concern paradigmatic alternatives of grammatical categories such as voice, number and case, but also groups of similar constructions, to which I refer as “construction families.”

## Construction Families

The term construction family is used in analogy to the notion of lexical family in the study of the mental lexicon, which is commonly characterized as an association network (Anderson, 1983; Dell, 1986). In order to explore the structure of this network, psycholinguists investigate how lexemes are accessed in online language use (Collins and Loftus, 1975; Anderson, 1983; Schreuder and Baayen, 1997).

Lexical access is a competition process that is determined by several factors. First, all else being equal, frequent items are more easily accessed or activated than infrequent ones (Forster and Chambers, 1973 among many others). Second, lexical access is facilitated by priming: if the target word is preceded by a lexical prime, it is more easily activated (Dell, 1986). And third, lexical access is crucially influenced by neighborhood density, which refers to the number of items that are phonetically and/or semantically similar to the target word. The word *cat*, for instance, has many phonetic neighbors, e.g., *rat, hat, vat, pat, mat, bat* and *at*, whereas *cup* has only a few, e.g., *cut, up*. Neighborhood density can slow down lexical access in word recognition tasks (Luce and Pisoni, 1998), but has facilitatory effects on the activation of lexemes in speech production (e.g., Dąbrowska, 2008) and word learning (e.g., Storkel, 2004). Taken together, these findings have led psychologists to characterize the mental lexicon as an activation network in which lexemes are grouped together into families of semantically and/or formally similar expressions (cf. 23).(23)

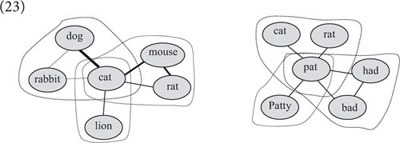

Like lexemes, constructions are organized in families of semantically or structurally similar grammatical patterns that influence each other in processing and acquisition (Diessel, 2019, p. 199–222). Construction families share some properties with paradigmatic alternatives such as active and passive sentences (see above). Yet, in contrast to the latter, the members of a construction family are only loosely associated with each other. They do not form tightly organized paradigms of grammatical categories such as voice and number, but are open-ended groups of constructions that do not (usually) exhibit the encoding asymmetries to which typologists refer as markedness. Consider, for instance, the following examples of the English resultative construction (cf. 24).

(24) a. John painted the door *red*.b. Bill broke the mirror *into pieces.*
  c. The lake froze *rock solid*.
  d. We drank the pub *dry*.
  e. John drank himself *sick*.

Resultative constructions designate an action that puts an NP argument into a particular state (Boas, 2003). Like many other argument-structure constructions, resultative constructions vary along several parameters. They generally include a resultative element, but this element can be an adjective or a prepositional phrase (24a–b). The verb is usually transitive, but there are also intransitive resultative constructions (24c). If the verb is transitive, the direct object may or may not be selected by the verb (24a–b vs. 24d). If the verb is intransitive, the construction either lacks a direct object (24c) or includes a “fake object,” usually a reflexive pronoun (24e) (Boas, 2003, p. 4–8). Considering this variation, Goldberg and Jackendoff (2004, p. 535) argued that resultatives do NOT form a “unified phenomenon” but “a sort of family of constructions.”

Like resultatives, copular clauses constitute a family of semantically and formally related constructions (e.g., Hengeveld, 1992 and Stassen, 1997). In English, for example, the copula *be* may be accompanied by a nominal, an adjective or a prepositional phrase (25a–c). If *be* is followed by a nominal, the copular clause may express identity (25a) or existence (25d); and if *be* is followed by an adjective, the copular clause expresses either a permanent state (25b) or a transitory event (25e) (which in some languages are formally distinguished by the use of different copular verbs, e.g., Spanish *ser* vs. *estar*).

(25) a. John is my friend.b. Bill is tall.
  c. The glass is on the table.
  d. There was an old man.
  e. Mary is tired.

Crucially, while the members of a construction family may be subsumed under a constructional schema, they are also horizontally related to one another. One piece of evidence for this comes from structural priming. Like lexemes, constructions prime each other (see Pickering and Ferreira, 2008 for a review). In the simplest case, the priming effect is caused by the prior use of the same construction. For instance, as Bock (1986) demonstrated in a pioneering study, speakers’ choice between the double-object construction (e.g., *Give me the money*) and the *to*-dative construction (e.g., *Give the money to me*) is crucially influenced by the prior use of these constructions. If the previous discourse includes a double-object construction, speakers tend to describe a scene depicting an act of transfer by a double-object construction, but if the previous discourse includes a *to*-dative construction, they are likely to describe the same scene by a the *to*-dative (cf. Bock and Griffin, 2000; Gries, 2005).

Crucially, structural priming does not only occur when prime and target have the same structure; it also occurs with distinct but similar constructions, suggesting that these constructions are related in speakers’ linguistic memory. For example, Bock and Loebell (1990) showed that sentences including a directional prepositional phrase prime the *to*-dative construction (cf. 26a–b), and Hare and Goldberg (2000) showed that sentences including a verb such as *provide (with)* prime the double-object construction (cf. 27a–b). In the first case, prime and target have similar structures but different meanings, and in the second case, they have similar meanings but different structures.

(26) a. The wealthy widow drove an old Mercedes to the church.b. The wealthy widow gave an old Mercedes to the church.
(27) a. The farmer provided the cows with something to eat.b. The farmer gave the cows something to eat.

Taken together, this research suggests that argument-structure constructions are organized in construction families with overlapping structural and/or semantic properties (cf. 28) similar to lexical expressions in the mental lexicon (see 23 above).(28)

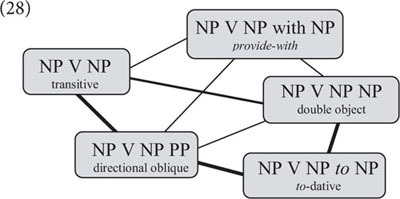

## Conclusion

To conclude, there is a large body of empirical results supporting the usage-based view of linguistic structure as a dynamic and emergent phenomenon. However, there is no consensus in the usage-based literature as to how the many results can be explained and integrated into a coherent model. In particular, the analysis of syntactic phenomena is unclear in this approach.

In this paper, I have argued that linguistic structure is best analyzed within a dynamic network model of grammar. The general idea has been expressed in previous studies. In fact, usage-based linguists seem to agree that grammar constitutes some kind of network (Langacker, 2008; Bybee, 2010). However, while the network view of grammar is frequently invoked in the usage-based literature, it has not yet been developed into an explicit theory or model.

In this paper, I have proposed network accounts for several core concepts of syntax including the notion of construction, grammatical word classes and constituent structure, which are commonly treated as primitive concepts of syntactic analysis. However, as we have seen, all of these concepts can be analyzed as emergent phenomena if they are construed as networks.

At the heart of the proposed analyses is a set of associative relations that concern different aspects of a speaker’s linguistic knowledge and that are shaped by various cognitive processes. Specifically, I have proposed the following set of relations:

1. Symbolic relations, which can be seen as pathways of semantic interpretation that arise when linguistic forms are routinely used to evoke a particular meaning.
2. Sequential relations, which are associations between linguistic elements in linear order that have developed into automated processing units.
3. Taxonomic relations, which specify hierarchical connections between lexical strings and constructional schemas at different levels of abstraction.
4. Filler-slot relations, which describe associations between individual slots of constructional schemas and particular lexical or phrasal fillers.
5. And constructional relations, which are lateral associations between similar or contrastive constructions that are grouped together in a family or paradigm^2^.

Taken together, the proposed relations provide a framework for the analysis of a wide range of grammatical phenomena as emergent concepts. In Diessel (2019), I have proposed additional network analyses for other grammatical phenomena and have discussed some of the topics of the current paper in more detail. Let me conclude with some general remarks on future research. There are many open questions, but here are three general points which, I believe, are of particular importance.

First, the various associative relations have different properties. For example, while one might assume that symbolic relations involve bidirectional associations between form and meaning, sequential relations are unidirectional in that sequential relations have an inherent forward direction, as evidenced by the fact that the language users anticipate upcoming elements in the speech stream. Each relation is influenced by particular cognitive processes and has specific properties that have to be investigated in more detail. This requires both experimental research and computational modeling. There are various computational frameworks using network models, but the conceptual and computational tools of Network Science appear to be particularly useful (Barabási, 2016). These tools have been used in psycholinguistic research on the mental lexicon (e.g., Vitevitch, 2008), but have not yet been used in research on grammar. Second, constructions and lexemes are the basic units of the grammar network. Construction grammar has emphasized the parallels between lexemes and constructions—both are commonly described as signs or symbols. Yet, in this paper, I have argued that constructions are best analyzed as networks and that the symbolic associations of constructions and lexemes have different properties. In my view, the notion of construction has to be revised in the context of a dynamic network model, but this needs careful consideration.

And third, the grammar network has been devised to account for dynamic processes in both language change and language acquisition. The latter comprises L1 acquisition and the acquisition of a second language. There are conspicuous parallels between language change and language acquisition (Diessel, 2011, 2012), but there are also differences between them. For instance, language change is influenced by social factors, such as prestige, which is of no or little importance to (early) L1 acquisition, but may have an impact on second language learning. Moreover, early L1 acquisition is a bottom-up process whereby children extract linguistic schemas from the ambient language (Gómez and Gerken, 1999); whereas language change typically involves the extension and modification of existing schemas, rather than the creation of entirely new ones; and L2 acquisition is influenced by interference from a learner’s native language (Diessel, 2019, p. 37–39). These differences raise questions about the general architecture of the grammar network and its status. Can we model language acquisition and language change within the same network or do we need two separate models to account for acquisition and change? Does L2 acquisition involve two separate networks or just one? And how do we account for language attrition in the context of a grammar network model?

## Data Availability Statement

All datasets generated for this study are included in the article/supplementary material, further inquiries can be directed to the corresponding author.

## Author Contributions

The author confirms being the sole contributor of this work and has approved it for publication.

## Conflict of Interest

The authors declare that the research was conducted in the absence of any commercial or financial relationships that could be construed as a potential conflict of interest.

## Footnotes

1 Like constructions, lexemes are horizontally related in the mental lexicon (e.g., Collins and Loftus, 1975). In order to account for the associations between lexemes, one might posit a particular type of “lexical link” in addition to the above relations (Diessel, 2019, p. 17–18, 200–202). However, lexical links play only a minor role in this paper.

2 In addition, one might posit a particular type of “lexical link” in order to account for horizontal associations between lexical items (see footnote 1).

## References

[B1] Abbot-Smith K. Tomasello M. (2006). Exemplar-learning and schematization in a usage-based account of syntactic acquisition. *Linguist. Rev.* 23 275–290.

[B2] Altmann G. T. M. Kamide Y. (1999). Incremental interpretation at verbs: restricting the domain of subsequent reference. *Cognition* 73 247–264. 10.1016/s0010-0277(99)00059-1 10585516

[B3] Anderson J. R. (1983). Retrieval of information from long-term memory. *Science* 220 25–30. 10.1126/science.6828877 6828877

[B4] Arnon I. Snider N. (2010). More than words: frequency effects for multi-word phrases. *J. Mem. Lang.* 62 67–82. 10.1016/j.jml.2009.09.005

[B5] Barabási A. L. (2016). *Network Science.* Cambridge: Cambridge University Press.

[B6] Baronchelli A. Ferrer-i-Cancho R. Paster-Satorras R. Chater N. Christiansen M. H. (2013). Networks in cognitive science. *Trends Cogn. Sci.* 17 348–360. 23726319 10.1016/j.tics.2013.04.010

[B7] Barsalou L. W. (1999). Perceptual symbol systems. *Behav. Brain Sci.* 22 577–660. 11301525 10.1017/s0140525x99002149

[B8] Barth D. Kapatsinski V. (2017). A multimodel inference approach to categorical variant choice: construction, priming and frequency effects on the choice between full and contracted forms of *am, are* and *is*. *Corpus Linguist. Linguist. Theory* 13 1–58.

[B9] Bates E. MacWhinney B. (1989). “Functionalism and the competition model,” in *The Crosslinguistic Study of Sentence Processing*, eds MacWhinney B. Bates E. (Cambridge: Cambridge University Press), 3–73.

[B10] Biber D. (2006). *University Language. A Corpus-Based Study of Spoken and Written registers.* Amsterdam: John Benjamins.

[B11] Boas H. C. (2003). *A Constructional Approach to Resultatives.* Stanford, CA: CSLI Publications.

[B12] Boas H. C. (2008). Determining the structure of lexical entries and grammatical constructions in construction grammar. *Annu. Rev. Cogn. Linguist.* 6 113–144. 10.1075/arcl.6.06boa

[B13] Bock J. K. (1986). Syntactic persistence in language production. *Cogn. Psychol.* 18 355–387. 10.1016/0010-0285(86)90004-6

[B14] Bock K. Griffin Z. (2000). The persistence of structural priming: transient activation or implicit learning? *J. Exp. Psychol. Gen.* 129 177–192. 10.1037/0096-3445.129.2.177 10868333

[B15] Bock K. Loebell H. (1990). Framing sentences. *Cognition* 35 1–39. 10.1016/0010-0277(90)90035-i 2340711

[B16] Bod R. (2009). From exemplar to grammar: a probabilistic analogy-based model of language learning. *Cogn. Sci.* 33 752–793. 10.1111/j.1551-6709.2009.01031.x 21585486

[B17] Bowerman M. (1988). “The ‘no negative evidence’ problem. How children avoid constructing an overgeneral grammar,” in *Explaining Language Universals*, ed. Hawkins J. A. (Oxford: Basil Blackwell), 73–101.

[B18] Bresnan J. Cueni A. Nikitina T. Baayen H. R. (2007). “Predicting the dative alternation,” in *Cognitive Foundations of Interpretation*, eds Boume G. Kraemer I. Zwarts J. (Amsterdam: Royal Netherlands Academy of Science), 69–94.

[B19] Bresnan J. Ford M. (2010). Predicting syntax: processing dative constructions in American and Australian varieties of English. *Language* 86 186–213.

[B20] Brooks P. Tomasello M. (1999). Young children learn to produce passives with nonce verbs. *Dev. Psychol.* 35 29–44. 10.1037/0012-1649.35.1.29 9923462

[B21] Brooks P. Tomasello M. Dodson K. Lewis L. B. (1999). Young children’s overgeneralizations with fixed transitivity verbs. *Child Dev.* 70 1325–1337. 10.1111/1467-8624.00097 10621959

[B22] Buchanan M. (2002). *Nexus. Small Worlds and the Groundbreaking Science of Networks.* New York, NY: W.W. Norton & Company.

[B23] Bybee J. (1995). Regular morphology and the lexicon. *Lang. Cogn. Process.* 10 425–455. 10.1080/01690969508407111

[B24] Bybee J. (2001). *Phonology and Language Use.* Cambridge: Cambridge University Press.

[B25] Bybee J. (2002). “Sequentiality as the basis of constituent structure,” in *The Evolution of Language out of Pre-Language*, eds Givón T. Malle B. F. (Amsterdam: John Benjamins), 109–132.

[B26] Bybee J. (2006). From usage to grammar: the mind’s response to repetition. *Language* 82 711–733. 10.1353/lan.2006.0186

[B27] Bybee J. (2010). *Language, Cognition, and Usage.* Cambridge: Cambridge University Press.

[B28] Bybee J. Hopper P. (eds.) (2001). *Frequency and the Emergence of Linguistic Structure.* Amsterdam: John Benjamins.

[B29] Bybee J. Modor C. L. (1983). Morphological classes as natural categories. *Language* 59 251–270. 10.2307/413574

[B30] Bybee J. Scheibman J. (1999). The effect of usage on degrees of constituency: the reduction of *don’t* in English. *Linguistics* 37 575–596.

[B31] Bybee J. Slobin D. I. (1982). Rules and schemas in the development of the English past tense. *Language* 58 265–289. 10.2307/414099

[B32] Chafe W. (1994). *Discourse, Consciousness, and Time. The Flow and Displacement of Conscious Experience in Speaking and Writing.* Chicago, IL: Chicago University Press.

[B33] Chang F. Dell G. S. Bock K. (2006). Becoming syntactic. *Psychol. Rev.* 113 234–272. 10.1037/0033-295x.113.2.234 16637761

[B34] Chen A. C.-H. (2020). Words, constructions and corpora: network representations of constructional semantics for Mandarin space particles. *Corpus Linguist. Linguist. Theory* Available online at: 10.1515/cllt-2020-0012 (accessed August 21, 2020).

[B35] Collins A. M. Loftus E. F. (1975). A spreading-activation theory of semantic processing. *Psychol. Rev.* 82 407–428. 10.1037/0033-295x.82.6.407

[B36] Croft W. (1991). *Syntactic Categories and Grammatical Relations. The Cognitive Organization of Information.* Chicago, IL: The University of Chicago Press.

[B37] Croft W. (2001). *Radical Construction Grammar.* Oxford: Oxford University Press.

[B38] Croft W. (2003). *Typology and Universals*, 2nd Edn Cambridge: Cambridge University Press.

[B39] Dąbrowska E. (2008). The effects of frequency and neighbourhood density on adult speakers’ productivity with Polish case inflections: an empirical test of usage-based approaches to morphology. *J. Mem. Lang.* 58 931–951. 10.1016/j.jml.2007.11.005

[B40] de Saussure F. (1916). *Course in General Linguistics.* LaSalle, IL: Open Court.

[B41] Dell G. S. (1986). A spreading-activation theory of retrieval in sentence production. *Psychol. Rev.* 93 283–321. 10.1037/0033-295x.93.3.283 3749399

[B42] Diessel H. (1997). “Verb-first constructions in German,” in *Lexical and Syntactical Constructions and the Construction of Meaning*, eds Verspoor M. Kee Dong L. Sweetser E. (Amsterdam: John Benjamins), 51–68. 10.1075/cilt.150.07die

[B43] Diessel H. (2007). Frequency effects in language acquisition, language use, and diachronic change. *New Ideas Psychol.* 25 108–127. 10.1016/j.newideapsych.2007.02.002

[B44] Diessel H. (2008). Iconicity of sequence. A corpus-based analysis of the positioning of temporal adverbial clauses in English. *Cogn. Linguist.* 19 457–482.

[B45] Diessel H. (2011). “Grammaticalization and language acquisition,” in *Handbook of Grammaticalization*, eds Heine B. Norrog H. (Oxford: Oxford University Press), 130–141.

[B46] Diessel H. (2012). “Language change and language acquisition,” in *Historical Linguistics of English: An International Handbook*, Vol. 2 eds Bergs A. Brinton L. (Berlin: Mouton de Gruyter), 1599–1613.

[B47] Diessel H. (2013). “Construction grammar and first language acquisition,” in *The Oxford Handbook of Construction Grammar*, eds Trousdale G. Hoffmann T. (Oxford: Oxford University Press), 347–364.

[B48] Diessel H. (2015). “Usage-based construction grammar,” in *Handbook of Cognitive Linguistics*, eds Dąbrowska E. Divjak D. (Berlin: Mouton de Gruyter), 295–321.

[B49] Diessel H. (2016). “Frequency and lexical specificity. A critical review,” in *Experience Counts: Frequency Effects in Language*, eds Behrens H. Pfänder S. (Berlin: Mouton de Gruyter), 209–237.

[B50] Diessel H. (2017). “Usage-based linguistics,” in *Oxford Research Encyclopedia of Linguistics*, ed. Aronoff M. (New York, NY: Oxford University Press). 10.1093/acrefore/9780199384655.013.363

[B51] Diessel H. (2019). *The Grammar Network. How Linguistic Structure is Shaped by Language Use.* Cambridge: Cambridge University Press 10.1017/9781108671040

[B52] Diessel H. Hilpert M. (2016). “Frequency effects in grammar,” in *Oxford Research Encyclopedia of Linguistics*, ed. Aronoff M. (New York, NY: Oxford University Press). 10.1093/acrefore/9780199384655.013.120

[B53] Diessel H. Tomasello M. (2005). A new look at the acquisition of relative clauses. *Language* 81 1–25. 10.1075/tilar.8.02kid 18416862

[B54] Eisenberg P. (2004). *Grundriss der Deutschen Grammatik.* Stuttgart: Metzler.

[B55] Elman J. L. (2009). On the meaning of words and dinosaur bones: lexical knowledge without a lexicon. *Cogn. Sci.* 33 1–36. 10.1515/9783111355191.1 19662108 PMC2721468

[B56] Elman J. L. Bates E. A. Johnson M. H. Karmiloff-Smith A. Parisi D. Plunckett K. (1996). *Rethinking Innateness. A Connectionist Perspective on Development.* Cambridge, MA: MIT Press.

[B57] Evans N. Levinson S. C. (2009). The myth of language universals: Language diversity and its importance for cognitive science. *Behav. Brain Sci.* 32 429–448. 10.1017/s0140525x0999094x 19857320

[B58] Evans N. Osada T. (2005). Mundari: the myth of a language without word classes. *Linguist. Typol.* 9 351–390.

[B59] Faulhaber S. (2011). *Verb Valency Patterns. A Challenge for Semantic-Based Accounts.* Berlin: Mouton de Gruyter.

[B60] Fine A. B. Jaeger T. F. Farmer T. A. Qian T. (2013). Rapid expectation adaptation during syntactic comprehension. *PLoS One* 8:e77661. 10.1371/journal.pone.0077661 24204909 PMC3813674

[B61] Forster K. I. Chambers S. M. (1973). Lexical access and naming time. *J. Verbal Learn. Verbal Behav.* 12 627–635. 10.1016/s0022-5371(73)80042-8

[B62] Fox B. A. Thompson S. A. (1990). A discourse explanation of the grammar of relative clauses in English conversations. *Language* 66 297–316.

[B63] Fox B. A. Thompson S. A. (2007). Relative clauses in English conversations. *Stud. Lang.* 31 293–326.

[B64] Frost R. Armstrong B. C. Christiansen M. H. (2019). Statistical learning research: a critical review and possible new directions. *Psychol. Bull.* 145 1128–1153. 10.1037/bul0000210 31580089

[B65] Garnsey S. M. Pearlmutter N. J. Myers E. E. Lotocky M. A. (1997). The contributions of verb bias and plausibility to the comprehension of temporarily ambiguous sentences. *J. Mem. Lang.* 7 58–93. 10.1006/jmla.1997.2512

[B66] Gerken L. A. (2006). Decisions, decisions: infant language learning when multiple generalizations are possible. *Cognition* 98 67–74. 10.1016/j.cognition.2005.03.003 15992791

[B67] Ghilardi M. F. Moisello C. Silvestri G. Ghez C. Krakauer J. W. (2009). Learning of a sequential motor skill comprises explicit and implicit components that consolidate differently. *J. Neurophysiol.* 101 2218–2229. 10.1152/jn.01138.2007 19073794 PMC2681421

[B68] Gobet F. P. Lane C. R. Croker S. Cheng P. C. H. Jones G. Oliver I. (2001). Chunking mechanisms in human learning. *Trends Cogn. Sci.* 5 236–243. 10.1016/s1364-6613(00)01662-4 11390294

[B69] Goldberg A. E. (1995). *Constructions. A Construction Grammar Approach to Argument Structure.* Chicago, IL: The University of Chicago Press.

[B70] Goldberg A. E. Jackendoff R. S. (2004). The English resultative as a family of constructions. *Language* 80 532–67. 10.1353/lan.2004.0129

[B71] Gómez R. L. (2002). Variability and detection of invariant structure. *Psychol. Sci.* 13 431–6. 10.1111/1467-9280.00476 12219809

[B72] Gómez R. L. Gerken L. A. (1999). Artificial grammar learning by 1-year-olds leads to specific and abstract knowledge. *Cognition* 70 109–135. 10.1016/s0010-0277(99)00003-7 10349760

[B73] Gordon P. C. Lowder M. W. (2012). Complex sentence processing: a review of theoretical perspectives on the comprehension of relative clauses. *Lang. Linguist. Compass* 6 403–415. 10.1002/lnc3.347

[B74] Greenberg J. H. (1966). *Language Universals, with Special Reference to Feature Hierarchies.* The Hague: Mouton.

[B75] Gries S. T. (2005). Syntactic priming: a corpus-based approach. *J. Psycholinguist. Res.* 34 365–99. 10.1007/s10936-005-6139-3 16142588

[B76] Gries S. T. Stefanowitsch A. (2004). Extending collexeme analysis. *Int. J. Corpus Linguist.* 9 97–129.

[B77] Hare M. Goldberg A. E. (2000). “Structural priming: purely syntactic?,” in *Proceedings of the 21st Annual Meeting of the Cognitive Science Society*, (London: Lawrence Erlbaum Associates), 208–211.

[B78] Haspelmath M. (2006). Against markedness (and what to replace it with). *J. Linguist.* 41 1–46.

[B79] Haspelmath M. (2008). Frequency vs. iconicity in explaining grammatical asymmetries. *Cogn. Linguist.* 19 1–33. 10.1515/cog.2008.001

[B80] Haspelmath M. Calude A. Spagnol M. Narrog H. Bamyaci E. (2014). Coding causal-noncausal verb alternations: a form–frequency correspondence explanation. *J. Linguist.* 50 587–625. 10.1017/s0022226714000255

[B81] Hay J. (2003). *Causes and Consequences of Word Structure.* New York, NY: Routledge.

[B82] Hay J. Baayen H. R. (2005). Shifting paradigms: gradient structure in morphology. *Trends Cogn. Sci.* 9 342–348. 10.1016/j.tics.2005.04.002 15993361

[B83] Hengeveld K. (1992). *Non-Verbal Predication: Theory, Typology, Diachrony.* Amsterdam: John Benjamins.

[B84] Hilpert M. (2014). *Construction Grammar and its Application to English.* Edinburgh: Edinburgh University Press.

[B85] Hoey M. (2005). *Lexical Priming. A New Theory of Words and Language.* London: Routledge.

[B86] Ibbotson P. (2020). *What it Takes to Talk: Exploring Developmental Cognitive Linguistics.* Berlin: Mouton de Gruyter.

[B87] Jackendoff R. (2002). *Foundations of Language. Brain, Meaning, Grammar, Evolution.* Oxford: Oxford University Press. 10.1017/s0140525x03000153 15377127

[B88] Jakobsen W. H. (1979). “Noun and verb in Nootkan,” in *Proceedings of the The Victoria Conference on Northwestern Languages November 4-5 1976*: British Columbia Provincial Museum, Heritage Record No. 4, ed. Efrat B. S. (Victoria, BC: British Columbia Provincial Museum), 83–153.

[B89] Jelinek E. (1995). “Quantification in Straits Salish,” in *Quantification in Natural Language*, eds Bach E. Jelinek E. Kratzer A. Partee B. (Dordrecht: Kluwer Academic Publisher), 487–540. 10.1007/978-94-011-0321-3_16

[B90] Johnson K. (1997). “Speech perception without speaker normalization. An exemplar model,” in *Talker Variability in Speech Processing*, eds Johnson K. Mullennix J. W. (San Diego, CA: Academic Press), 145–165.

[B91] Kamide Y. Altmann G. T. M. Haywood S. L. (2003). The time-course of prediction in incremental sentence processing: evidence from anticipatory eye movements. *J. Mem. Lang.* 49 133–156. 10.1016/s0749-596x(03)00023-8

[B92] Keenan E. L. Dryer M. S. (2007). “Passive in the world’s languages,” in *Language Typology and Syntactic Description*. *Complex Constructions*, 2nd Edn, Vol. 1 ed. Shopen T. (Cambridge: Cambridge University Press), 325–361.

[B93] Krug M. (1998). String frequency. A cognitive motivating factor in coalescence, language processing, and linguistic change. *J. Engl. Linguist.* 26 286–320. 10.1177/007542429802600402

[B94] Kuperberg G. R. Jaeger T. F. (2016). What do we mean by prediction in language comprehension. *Lang. Cogn. Neurosci.* 31 32–59. 10.1080/23273798.2015.1102299 27135040 PMC4850025

[B95] Kurumada C. Jaeger T. F. (2015). Communicative efficiency in language production: optional case-marking in Japanese. *J. Mem. Lang.* 83 152–178. 10.1016/j.jml.2015.03.003

[B96] Langacker R. W. (1991). *Concept, Image, and Symbol. The Cognitive Basis of Grammar.* Berlin: Mouton de Gruyter.

[B97] Langacker R. W. (1997). Constituency, dependency, and conceptual grouping. *Cogn. Linguist.* 8 1–32. 10.1515/cogl.1997.8.1.1

[B98] Langacker R. W. (2008). *Cognitive Grammar. A Basic Introduction.* Oxford: Oxford University Press.

[B99] Levelt W. J. M. (1989). *Speaking: From Intention to Articulation.* Cambridge, MA: MIT Press.

[B100] Levin B. Rappaport Hovav M. (2005). *Argument Realization.* Cambridge: Cambridge University Press.

[B101] Levy R. (2008). Expectation-based syntactic comprehension. *Cognition* 106 1126–1177. 10.1016/j.cognition.2007.05.006 17662975

[B102] Logan G. D. (1988). Towards an instance theory of automatization. *Psychol. Rev.* 95 492–527. 10.1037/0033-295x.95.4.492

[B103] Lorenz D. Tizón-Couto D. (2017). Coalescence and contraction of V-*to*-V_*inf*_ sequences in American English – evidence from spoken language. *Corpus Linguist. Linguist. Theory.* Available online at: 10.1515/cllt-2015-0067 (accessed March 30, 2017).

[B104] Luce P. A. Pisoni D. P. (1998). Recognizing spoken words: the neighborhood activation model. *Ear Hear.* 19 1–36. 10.1097/00003446-199802000-00001 9504270 PMC3467695

[B105] MacDonald M. C. Pearlmutter N. J. Seidenberg M. S. (1994). Lexical nature of syntactic ambiguity resolution. *Psychol. Rev.* 101 676–703. 10.1037/0033-295x.101.4.676 7984711

[B106] MacWhinney B. (ed.) (1999). *The Emergence of Language.* Mahwah, NJ: Lawrence Erlbaum.

[B107] Mak W. M. Vonk W. Schriefers H. (2006). Animacy in processing relative clauses. *J. Mem. Lang.* 54 466–490. 10.1016/j.jml.2006.01.001

[B108] Norde M. Morris C. (2018). “Derivation without category change. A network-based analysis of diminutive prefixoids in Dutch,” in *Category Change from a Constructional Perspective*, eds van Goethem K. Norde M. Coussé E. Vanderbauwhede G. (Amsterdam: John Benjamins), 47–92. 10.1075/cal.20.03nor

[B109] Pickering M. J. Ferreira V. S. (2008). Structural priming: a critical review. *Psychol. Bull.* 134 427–459. 10.1037/0033-2909.134.3.427 18444704 PMC2657366

[B110] Pierrehumbert J. B. (2001). “Exemplar dynamics: word frequency, lenition and contrast,” in *Frequency and the Emergence of Linguistic Structure*, eds Bybee J. Hopper P. (Amsterdam: John Benjamins), 137–158. 10.1075/tsl.45.08pie

[B111] Pinker S. (1989). *Learnability and Cognition. The Acquisition of Argument Structure.* Cambridge, MA: MIT Press.

[B112] Reali F. Christiansen M. H. (2007). Processing of relative clauses is made easier by frequency of occurrence. *J. Mem. Lang.* 57 1–23. 10.1016/j.jml.2006.08.014

[B113] Roland D. Dick F. Elman J. L. (2007). Frequency of basic English grammatical structures: a corpus analysis. *J. Mem. Lang.* 57 348–379. 10.1016/j.jml.2007.03.002 19668599 PMC2722756

[B114] Roland D. Mauner G. O’Meara C. Yun H. (2012). Discourse expectations and relative clause processing. *J. Mem. Lang.* 66 479–508. 10.1016/j.jml.2011.12.004

[B115] Rumelhart D. E. McClelland J. L. (1986). “On learning the past tenses of English verbs,” in *Parallel Distributed Processing: Explorations in the Microstructure of Cognition*, Vol. 2 eds Rumelhart D. E. McClelland J. L. The PDP Research Group (Cambridge, MA: MIT Press), 216–271.

[B116] Schmid H. J. (2020). *The Dynamics of the Linguistic System: Usage, Conventionalization, and Entrenchment.* Oxford: Oxford University Press.

[B117] Schreuder R. Baayen H. R. (1997). How complex simplex words can be. *J. Mem. Lang.* 37 118–139. 10.1006/jmla.1997.2510

[B118] Siew C. S. Q. Wulff D. U. Beckage N. M. Kenett Y. N. (2019). Cognitive network science: a review of research on cognition through the lens of network representations, processes, and dynamics. *Complexity* 1919 1–24. 10.1155/2019/2108423

[B119] Spivey-Knowlton M. J. Sedivy J. (1995). Resolving attachment ambiguities with multiple constraints. *Cognition* 55 227–267. 10.1016/0010-0277(94)00647-4 7634760

[B120] Sporns O. (2011). *Networks of the Brain.* Cambridge, MA: MIT Press.

[B121] Stassen L. (1997). *Intransitive Predication.* Oxford: Oxford University Press.

[B122] Storkel H. L. (2004). Do children acquire dense neighborhoods? An investigation of similarity neighborhoods in lexical acquisition. *Appl. Psycholinguist.* 25 201–221. 10.1017/s0142716404001109

[B123] Szmrecsanyi B. Hinrichs L. (2008). “Probabilistic determinants of genitive variation in spoken and written English. A multivariate comparison across time, space, and genres,” in *The Dynamics of Linguistic Variation: Corpus Evidence on English Past and Present*, eds Nevalainen T. Taavitsainen I. Pahta P. Korhonen M. (Amsterdam: John Benjamins), 291–309. 10.1075/silv.2.22szm

[B124] Talmy L. (2000). *Toward a Cognitive Semantics, Concept Structuring Systems*, Vol. 1 Cambridge, MA: MIT Press.

[B125] Thelen E. Smith L. B. (1995). *A Dynamic Systems Approach to the Development of Cognition and Action.* Cambridge, MA: MIT Press. 10.1162/jocn.1995.7.4.512 23961909

[B126] Tomasello M. (2003). *Constructing a Language. A Usage-Based Approach.* Cambridge, MA: Harvard University Press.

[B127] Tomasello M. Brooks P. (1998). Young children’s earliest transitive and intransitive constructions. *Cogn. Linguist.* 9 379–395. 10.1515/cogl.1998.9.4.379

[B128] Trueswell J. C. (1996). The role of lexical frequency in syntactic ambiguity resolution. *J. Mem. Lang.* 35, 566–585. 10.1006/jmla.1996.0030

[B129] Tulving E. Schacter D. L. (1990). Priming and human memory systems. *Science* 247 301–306. 10.1126/science.2296719 2296719

[B130] Van de Velde F. (2014). “Degeneracy: the maintenance of constructional networks,” in *The Extending Scope of Construction Grammar*, eds Boogaart R. Colleman T. Rutten G. (Berlin: Mouton de Gruyter), 141–179.

[B131] van Trijp R. (2010). Grammaticalization and semantic maps: evidence from artificial language evolution. *Linguist. Discov.* 8 310–326.

[B132] Vitevitch M. S. (2008). What can graph theory tell us about word learning and lexical retrieval. *J. Speech Lang. Hear. Res.* 51 408–9. 10.1044/1092-4388(2008/030) 18367686 PMC2535910

[B133] Weiner E. J. Labov W. (1983). Constraints on agentless passive. *J. Linguist.* 19 29–58. 10.1017/s0022226700007441

[B134] Wolk C. Joan B. Rosenbach A. Szmrecsanyi B. (2013). Dative and genitive variability in Late Modern English: exploring cross-constructional variation and change. *Diachronica* 30 382–419. 10.1075/dia.30.3.04wol

[B135] Wray A. (2002). *Formulaic Language and the Lexicon.* Cambridge: Cambridge University Press.

